# UNDERSTANDING CHALLENGES OF PERSONS WITH COGNITIVE IMPAIRMENTS THROUGH THEIR LIVED EXPERIENCES

**DOI:** 10.1093/geroni/igac059.2094

**Published:** 2022-12-20

**Authors:** Maurita Harris, Elizabeth Lydon, Widya Ramadhani, Raksha Mudar, Wendy Rogers

**Affiliations:** University of Illinois Urbana-Champaign, Champaign, Illinois, United States; University of Illinois Urbana-Champaign, Champaign, Illinois, United States; University of Illinois Urbana-Champaign, Urbana, Illinois, United States; University of Illinois Urbana Champaign, Champaign, Illinois, United States; University of Illinois Urbana Champaign, Champaign, Illinois, United States

## Abstract

Older adults aging with cognitive impairments face a variety of challenges related to their memory, thinking, and concentration in their everyday activities. Understanding their lived experiences s critical to inform the development of technology and supports that can help everyday activities and improve quality of life. We have designed an in-depth interview study to explore the everyday challenges of older adults with cognitive impairments and their response strategies. We will present two case studies to illustrate the richness of the data and its value for guiding intervention design: (1) one older adult with a post-stroke cognitive impairment (PSCI) and (2) one older adult with mild cognitive impairment (MCI). As expected, these individuals reported challenges in different functional activities and described varying solution strategies. The older adult with PSCI noted challenges with completing steps and remembering things when engaging with technology-mediated social activities. This individual reported responding to these challenges by having their own method, such as writing things down or receiving assistance from others. Challenges the older adult with MCI experienced when engaging with technology-mediated social activities, long-distance travel, and caregiving were planning, completing steps, remembering things, and experiencing emotions. This individual responded to their challenges by developing visualizations, methods, routine, and receiving assistance from others. These initial insights about the range of challenges with everyday activities and response strategies highlight the value of qualitative needs assessments in understanding the needs of those aging with cognitive impairment to guide future technology and support.

